# Supplementary material for: Postpartum versus postnatal period: Do the name and duration matter?
Source: PLoS One. 2024 Apr 26;19(4):e0300118. doi: 10.1371/journal.pone.0300118 (PMC11051636; doi:10.1371/journal.pone.0300118)
Supplement: S1 File — (PDF) [file pone.0300118.s001.pdf]

S1 File

Search Strategy

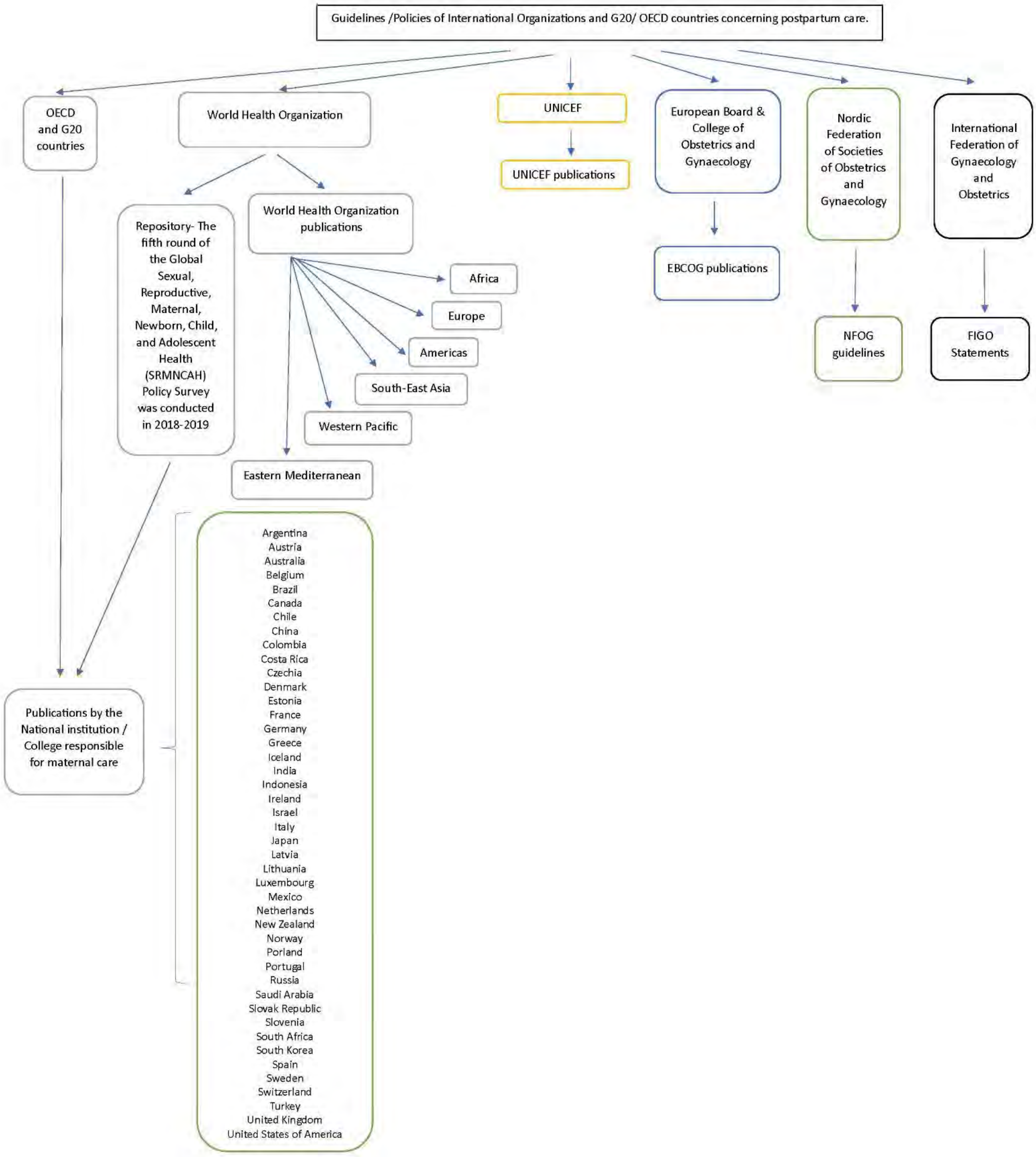

Supplementary Figure 1: Types of Resources included to conduct the scoping review on guidelines on routine postpartum care by international organisations, G20 and OECD countries*.

*OECD- Organisation for Economic Co-operation and Development, G 20- Group of 20, FIGO- International Federation of Gynaecology and Obstetrics, UNICEF- United Nations Children's Fund, EBCOG- European Board & College of Obstetrics and Gynaecology, NFOG- Nordic Federation of Societies of Obstetrics and Gynaecology

Supplementary Table 1.1: Search strategy to locate documents on routine postpartum care by different international organisations, G20 and OECD countries*.

**Organization**		**Search terms**
**WHO Repository- The fifth round of the Global Sexual, Reproductive, Maternal, Newborn, Child, and Adolescent Health (SRMNCAH Policy Survey)** (13)		
1	Postpartum	'Postpartum' AND 'Country'<br>Filters-Maternal and Newborn; English
2	Postnatal	'Postnatal' AND 'Country'<br>Filters-Maternal and Newborn; English
3	Interpregnancy	'Interpregnancy' AND 'Country'<br>Filters-Maternal and Newborn; English
4	Interconception	'Interconception' AND 'Country'<br>Filters-Maternal and Newborn; English
5	Maternal	'Maternal' AND 'Country'<br>Filters-Maternal and Newborn; English
**WHO**		
1	WHO publications	Keywords- 'Maternal heath', 'Postpartum'<br>Publication type- Country Cooperation Strategy; Governing Bodies Documentation, Guidance, Guideline, handbook, Manual, Policy Brief, Meeting Report, Technical documentation, Standards<br>Publishing Offices-Any<br>Countries/Areas-Any<br>Year-Any
**UNICEF**		
1	UNICEF publications	Filters-Guidance; Publications; Maternal health; Newborn care
**FIGO**		
1	FIGO Statements	Filters-Maternal Health; Newborn Health
**European Board & College of Obstetrics and Gynaecology (EBCOG)**		
1	EBCOG publications	Keywords-'Maternal', 'Postpartum', 'Postnatal', 'Interpregnancy', 'Interconception'
**Nordic Federation of Societies of Obstetrics and Gynaecology**		
1	Clinical Guidelines and Nordic Collaboration presented by NFOG	N/A
**National Guidelines**		
1.	Publications by the National Institution / College responsible for maternal care	Keywords-'Maternal', 'Postpartum', 'Postnatal', 'Interpregnancy', 'Interconception'

*OECD- Organisation for Economic Co-operation and Development, G 20- Group of 20, WHO- World Health Organization, FIGO- International Federation of Gynaecology and Obstetrics, UNICEF- United Nations Children's Fund, EBCOG- European Board & College of Obstetrics and Gynaecology

Supplementary Table 1.2: Available guidelines/policies, frameworks among eligible organisations and countries

Country	Number of documents (In all languages)	Number of documents Published in English*	Number of documents included in the review
WHO	49	49	10
UNICEF	4	4 (One duplicate with WHO)	0
European Board & College of Obstetrics and Gynaecology	2	2	1
The Nordic Federation of Societies of Obstetrics and Gynecology	0	0	0
FIGO	0	0	0
Argentina	2	0	0
Austria	1	0	0
Australia	4	4	1
Belgium	2	1	0
Brazil	1	0	0
Canada	1	1	1
Chile	0	0	0
China	2	0	0
Colombia	0	0	0
Costa Rica	0	0	0
Czechia	0	0	0
Denmark	1	0	0
Estonia	0	0	0
France	2	1	0
Germany	2	0	0
Greece	0	0	0
Iceland	0	0	0
India	7	7	5
Indonesia	1	0	0
Ireland	0	0	0
Israel	0	0	0
Italy	1	0	0
Japan	1	0	0
Latvia	0	0	0
Lithuania	0	0	0
Luxembourg	0	0	0
Mexico	8	0	0
Netherlands	1	0	0
New Zealand	0	0	0
Norway	1	0	0
Porland	0	0	0
Portugal	2	0	0
Russia	1	0	0
Saudi Arabia	1	0	0
Slovak Republic	0	0	0

Slovenia	0	0	0
South Africa	5	4	4
South Korea	1	0	0
Spain	1	0	0
Sweden	3	0	0
Switzerland	1	0	0
Turkey	1	0	0
United Kingdom	2	2	2
United States of America	2	2	2

WHO- World Health Organization, UNICEF- United Nations Children's Fund; *Several documents were omitted from the review either because they were restricted to a specific disease, or subtopic related to the postpartum period (e.g., guidelines only addressing postpartum haemorrhage, gestational diabetes, etc.) or could not be considered as a guideline or a strategy (e.g., Desk review, situation analysis, etc.)
