# Supplementary material for: Postpartum versus postnatal period: Do the name and duration matter?
Source: PLoS One. 2024 Apr 26;19(4):e0300118. doi: 10.1371/journal.pone.0300118 (PMC11051636; doi:10.1371/journal.pone.0300118)
Supplement: S1 Table — (PDF) [file pone.0300118.s002.pdf]

S1 Table: Summary of findings on routine postpartum care published by international organisations, G20 and OECD countries*

International body/country and Institution	Year of publication of the latest guideline/ Position Paper/ Strategy	Use of 'Postpartum' or 'Postnatal' term to refer to care/ services for the mother following delivery*		Postpartum length	Service length	Service frequency
		Postpartum	Postnatal			
**Europe [1]**<br>Obstetrics and Gynaecology European Board & College Obstetric and Neonatal Servicess	**2014**<br>Obstetric and Neonatal Services Standards of Care for Women's Health in Europe	+	+	Not given	Not given	Not given
**WHO [2]**	**2022**<br>WHO recommendations on maternal and newborn care for a positive postnatal experience	+	+	6 weeks	6 weeks	Within 24 hours<br>On day 3 (48-72 hours)<br>Between day 7 and 14<br>6 weeks
**WHO [3]**	**2015**<br>Pregnancy, Childbirth, Postpartum and Newborn Care: A guide for essential practice	+	+	Not given	6 weeks	Within 24 hours after childbirth.<br>Day 3 (48-72 hours)<br>Between day 7 and 14<br>Clinic visit: 6 weeks
**WHO [4]**	**2013**<br>Counselling for maternal and newborn health care: a handbook for building skills	+	+	Not given	6 weeks	Within 24 hours, first visit (could be a home visit)<br>Within 1 week, preferably on day 3<br>Second visit 7-14 days after birth<br>Third visit 4-6 weeks after birth

International body/country and Institution	Year of publication of the latest guideline/ Position Paper/ Strategy	Use of 'Postpartum' or 'Postnatal' term to refer to care/ services for the mother following delivery*		Postpartum length	Service length	Service frequency
		Postpartum	Postnatal			
**WHO [5]**	**2013**<br>WHO recommendations on Postnatal care of the mother and newborn	+	+	Days and weeks following child birth	6 weeks	Within 24 hours<br>Day 3 (48-72 hours)<br>7–14 days<br>6 weeks
**WHO [6]**	**2010**<br>WHO Technical Consultation on Postpartum and Postnatal Care	+	+	6 weeks	6 weeks	Within one hour<br>72 hours<br>10–14 days<br>6–8 weeks
**WHO [7]**	**2009**<br>WHO Recommended Interventions for Improving Maternal and Newborn Health	+	+	Not given	6 weeks	Not given
**WHO [8]**	**2003**<br>Pregnancy, Childbirth, Postpartum and Newborn Care: A guide for essential practice	+	+	Not given	6 weeks	Within 1st week, preferably within 2-3 days<br>4-6 weeks
**WHO [9]**	**1998**<br>Postpartum care of the mother and newborn : a practical guide	+	+	6 weeks	6 weeks	6 hours (6-12 hrs)<br>6 days (3-6 days)<br>6 weeks<br>6 months
**WHO [10]**	**1996**<br>Mother-Baby Package: Implementing safe motherhood in countries	+	-	Not given	Not given	Not given

International body/country and Institution	Year of publication of the latest guideline/ Position Paper/ Strategy	Use of ‘Postpartum’ or ‘Postnatal’ term to refer to care/ services for the mother following delivery*		Postpartum length	Service length	Service frequency
		Postpartum	Postnatal			
**WHO [11]**	**1976**<br>New Trends and Approaches in the Delivery of Maternal and Child Care in Health Services	-	+	Not given	Not given	not given
**Australia [12]**<br>Council of Australian Governments	**2019**<br>Woman-centred care Strategic directions for Australian maternity services	-	+	Not given	52 weeks	Not given
**Canada [13]**<br>Public Health Agency of Canada	**2020**<br>Family-Centred Maternity and Newborn Care: National Guidelines	+	+	Not given	6 weeks	National uniformity is not present with each province determining its postpartum care plan
**India [14]**<br>National Rural Health Mission	**2021**<br>Induction Training Module for ASHAs	-	+	Not given	6 weeks	For home delivery visits on days - 1, 3, 7, 14, 21, 28, and 42.<br>For institutional delivery visits on days - 3, 7, 14, 21, 28, and 42
**India [15]**<br>Ministry of Health and Welfare	**2018**<br>Guidelines on midwifery services in India 2018	+	+	Not given	6 weeks	Not given
**India [16]**<br>Maternal Health Division Ministry of Health & Family Welfare Government of India	**2013**<br>Maternal and Newborn Health Toolkit	+	+	Not given	Not given	Not given

International body/country and Institution	Year of publication of the latest guideline/ Position Paper/ Strategy	Use of 'Postpartum' or 'Postnatal' term to refer to care/ services for the mother following delivery*		Postpartum length	Service length	Service frequency
		Postpartum	Postnatal			
**India [17]**<br>Maternal Health Division<br>Ministry of Health and Family Welfare<br>Government of India	**2010**<br>Guidelines for Antenatal Care and Skilled Attendance at Birth	+	+	Not given	6 weeks	First visit 1st day (within 24 hours)<br>Second visit 3rd day after delivery<br>Third visit 7th day after delivery<br>Fourth visit 6 weeks after delivery
**India [18]**<br>Ministry of Health and Welfare	**2009**<br>Trainers' Guide for Training of Medical Officers in Pregnancy Care and Management of Common Obstetric Complications	+	+	Not given	Not given	Not given
**South Africa [19]**<br>Department of Health	**2021**<br>South African maternal, perinatal, and neonatal Health Policy	+	+	Not given	Not given	Not given
**South Africa [20]**	**2023**<br>Saving Mothers and Babies 2017-2019:<br>Executive Summary	+	+	Not given	Not given	Not given
**South Africa [21]**<br>Department of Health	**2016**<br>Guidelines for Maternity Care	+	+	Not given	6 weeks	Immediately<br>3 to 6 days<br>6 weeks
**South Africa [22]**<br>Department of Health	**N/A**<br>Maternal Child and Women's Health Draft Policy Document	+	+	Not given	Not given	Not given

International body/country and Institution	Year of publication of the latest guideline/ Position Paper/ Strategy	Use of 'Postpartum' or 'Postnatal' term to refer to care/ services for the mother following delivery*		Postpartum length	Service length	Service frequency
		Postpartum	Postnatal			
**United Kingdom [23]**<br>NICE & The Royal College of Obstetricians and Gynaecologists	**2021**<br>Postnatal care	+	+	Not given	8 weeks	Within 36 hours after discharge<br>Health worker home visit within 7-14 days<br>6-8 weeks by GP
**United Kingdom [24]**<br>NICE & The Royal College of Obstetricians and Gynaecologists	**2006**<br>Postnatal care up to 8 weeks after birth	-	+	Not given	8 weeks	6-8 weeks health screening
**United States of America [25]**<br>White House	**2022**<br>White House blueprint for addressing the maternal health crisis	+	+	Not given	Not given	Not given
**United States of America [26]**<br>American College of Obstetricians and Gynecologists	**2018**<br>Optimizing Postpartum Care	+	-	Not given	12 weeks	Blood pressure check- 3-10 days<br>High-risk contacts: 1-3 weeks<br>If necessary: 3-12 weeks<br>4-12 weeks- comprehensive postpartum visit

*OECD- Organisation for Economic Co-operation and Development, G 20- Group of 20, FIGO- International Federation of Gynaecology and Obstetrics, WHO- World Health Organisation
No : -   Yes : +

References for Supplementary Table 1

1. Obstetrics and Gynaecology European Board and College of Obstetric and Neonatal Services. Standards of Care for Obstetric and Neonatal Services. Belgium: Obstetrics and Gynaecology European Board and College of Obstetric and Neonatal Services; 2014. https://eeca.unfpa.org/en/publications/EuropeanSRHStandardsRU
2. World Health Organization. WHO recommendations on maternal and newborn care for a positive postnatal experience. Geneva: World Health Organization; 2022. https://www.who.int/publications/i/item/9789240045989
3. World Health Organization. Pregnancy, Childbirth, Postpartum and Newborn Care: A guide for essential practice. 3 ed. Geneva: World Health Organization & UNICEF; 2015. https://www.who.int/publications/i/item/9789241549356
4. Department of Maternal Newborn Child and Adolescent Health. Counselling for maternal and newborn health care: a handbook for building skills. Geneva: World Health Organization; 2013. https://apps.who.int/iris/handle/10665/44016
5. World Health Organization. WHO recommendations on Postnatal care of the mother and newborn. Geneva: World Health Organization; 2013. https://apps.who.int/iris/handle/10665/97603
6. Department of Making Pregnancy Safer. WHO Technical Consultation on Postpartum and Postnatal Care. Geneva: World Health Organization; 2010. https://apps.who.int/iris/handle/10665/70432
7. Department of Making Pregnancy Safer. WHO Recommended Interventions for Improving Maternal and Newborn Health. 2 ed. Geneva: World Health Organization; 2009. https://apps.who.int/iris/bitstream/handle/10665/69509/WHO_MPS_07.05_eng.pdf;sequence=1
8. World Health Organization. Pregnancy, Childbirth, Postpartum and Newborn Care: A guide for essential practice. 1 ed. Geneva: World Health Organization; 2003. https://www.jica.go.jp/Resource/project/philippines/0600894/04/pdf/pcpnc_who.pdf
9. World Health Organization. Postpartum care of the mother and newborn : a practical guide. Geneva: World Health Organization; 1998. https://apps.who.int/iris/handle/10665/66439
10. Division of Family Health. Mother-Baby Package: Implementing safe motherhood in countries. Geneva: World Health Organization; 1996. https://www.who.int/publications/i/item/WHO-FHE-MSM-94.11-Rev.1
11. World Health Organization. New Trends and Approaches in the Delivery of Maternal and Child Care in Health Services. Geneva: World Health Organization; 1976. https://apps.who.int/iris/handle/10665/41219
12. Department of Health and Aged Care. Woman-centred care, Strategic directions for Australian maternity services. Canberra: Council of Australian Governments 2019. https://www.health.gov.au/resources/publications/woman-centred-care-strategic-directions-for-australian-maternity-services?language=und
13. Public Health Agency of Canada. Chapter 5 Postpartum Care. Family-Centred Maternity and Newborn Care National Guidelines. Ottawa: Public Health Agency of Canada; 2020: 1-75. https://www.canada.ca/en/public-health/services/maternity-newborn-care-guidelines.html
14. National Rural Health Mission. Induction Training Module for ASHAs. New Delhi: National Rural Health Mission; 2021. https://nhm.gov.in/images/pdf/communitisation/asha/ASHA_Induction_Module_English.pdf
15. National Health Mission. Guidelines on Midwifery Services in India. New Delhi: Ministry of Health and Family Welfare; 2018. https://nhm.gov.in/New_Updates_2018/NHM_Components/RMNCHA/MH/Guidelines/Guidelines_on_Midwifery_Services_in_India.pdf
16. Maternal Health Division. Maternal and Newborn Health Toolkit. New Delhi: Ministry of Health and Family Welfare; 2013. http://www.dkhfw.in/wp-content/uploads/2017/07/Maternal_Newborn_Health_Toolkit.pdf
17. Maternal Health Division. Guidelines for Antenatal Care and Skilled Attendance at Birth. New Delhi: Ministry of Health and Family Welfare; 2010. https://nhm.gov.in/images/pdf/programmes/maternal-health/guidelines/sba_guidelines_for_skilled_attendance_at_birth.pdf
18. Maternal Health Division. Trainers' Guide for Training of Medical Officers in Pregnancy Care and Management of Common Obstetric Complications. New Delhi: Ministry of Health and Family Welfare; 2009. https://nhm.gov.in/images/pdf/programmes/maternal-health/guidelines/trainees_guide_for_training_of_mos_in_preg_care.pdf
19. South African Maternal, Perinatal and Neonatal Health Policy. Pretoria: South African National Department of Health; 2021. https://knowledgehub.health.gov.za/elibrary/south-african-maternal-perinatal-and-neonatal-health-

policy#:~:text=The%20policy%20focusses%20on%20the,access%20to%20post%2Ddelivery%20contraception.
20. Saving Mothers and Babies 2017-2019: Executive Summary. Pretoria: National Department of Health; 2023. https://www.health.gov.za/wp-content/uploads/2023/05/SAVING-MOTHERS-SAVING-BABIES-REPORT-2017-2019.pdf
21. Department of Health Republic of South Africa. Guidelines for Maternity Care. 4 ed. South Africa: Department of Health, Republic of South Africa; 2016. https://knowledgehub.health.gov.za/elibrary/guidelines-maternity-care-south-africa-2016
22. Maternal Child and Women's Health Draft Policy Document. Pretoria: Department of Health. https://www.gov.za/sites/default/files/gcis_document/201409/childwomenhealth0.pdf
23. National Institute for Health Care Excellence and The Royal College of Obstetricians and Gynaecologists. Postnatal care. United Kingdom: National Institute for Health and Care Excellence and The Royal College of Obstetricians and Gynaecologists; 2021. https://www.nice.org.uk/guidance/ng194
24. Postnatal care up to 8 weeks after birth. London: National Institute for Health and Care Excellence; 2006. https://www.northernlms.org/wp-content/uploads/2020/04/NICE-Guidance-Postnatal-Care.pdf
25. White House Blueprint for Addressing the Maternal Health Crisis. Washington: The White House; 2022. https://www.whitehouse.gov/wp-content/uploads/2022/06/Maternal-Health-Blueprint.pdf
26. Stuebe A, Auguste T, Gulati M. Optimizing postpartum care. ACOG Committee Opinion No. 736. *Obstetrics & Gynecology* 2018; **131**(5): e140. https://www.acog.org/clinical/clinical-guidance/committee-opinion/articles/2018/05/optimizing-postpartum-care
