# Supplementary material for: Postpartum versus postnatal period: Do the name and duration matter?
Source: PLoS One. 2024 Apr 26;19(4):e0300118. doi: 10.1371/journal.pone.0300118 (PMC11051636; doi:10.1371/journal.pone.0300118)
Supplement: Attachment — Submitted filename: Response to Reviewers.docx [file pone.0300118.s003.docx]

12th January 2024

Dr Steve Zimmerman

Senior Editor

PLOS ONE

Dear Dr Zimmerman

**Postpartum versus postnatal period: Do the name and duration matter? (PONE-D-23-34077)**

We were pleased to have an opportunity to revise our manuscript entitled “Postpartum versus postnatal period: Do the name and duration matter?” We appreciate the constructive feedback provided by the reviewers and believe that their suggestions have improved the manuscript further. Our responses to the reviewers’ comments are given below along with line numbers where we have made changes in the manuscript (shown as track changes in the manuscript).

Additionally, to ensure the currency of work, we have updated the Web of Science search on the use of the terms ‘postpartum and ‘postnatal’ with 2023 data (originally presented as 2022 data) on lines 225-227.

|  | **Comment** | **Response** |
| --- | --- | --- |
| **Reviewer One** | | |
| 1 | In your results, you say the initial search yielded 57 documents from 28 countries, but with the additional search, you added one more country-level document. However, in the following statement, you then say the search yield 58 documents from 32 countries. Why is there a discrepancy in countries available from 28 to 32 but you only added one country? | We found 57 documents from 28 countries including UK. Following bibliographic search of these documents, we were able to identify additional guideline from UK. Therefore, 2 documents were included from UK (Supplementary table 1). Thus, final count should be 58 documents from 28 countries. It was an error stating number of countries as 32 (Supplementary File 1). Thank you for highlighting the mistake.  We have corrected it as 28 countries.(lines 127 & 128) |
| 2 | Please spell out UK and US at first use (line 129) | We have spelled out UK and USA at first use. |
| 3 | Canada has provinces not states (line 193 and table) | Thank you highlighting the error.  We changed from” state” to “province” in line 196 and at relevant places in the Tables. |
| **Reviewer Two** | | |
| 1 | Although terms postpartum and postnatal are used interchangeably by some as the authors state, in the abstract the focus is on the term postpartum in the results section | Thank you for the concern. We acknowledge the fact that the use of postpartum term in the results section in the abstract may be interpreted as bias towards “postpartum” term. Therefore, we have replaced the postpartum term with “after delivery” or included both postpartum and postnatal terms in “results” and “conclusion” sections in the abstract. (lines 45,47, 53 and 54). |
| 2 | Title - Does the…. | ‘Does’ represents singular concept whereas ‘Do’ represents plural. We have used two concepts in the title: name and duration, therefore, ‘Do’ is grammatically correct. We have confirmed this with our English Instructors. |
| 3 | Line 150 - From several recent WHO publications, it looks like they use the term postnatal instead of postpartum pretty consistently. I believe stating this here would provide some additional clarity | We understand your point, however, line 150 is in the results section. Results are from the Guideline documents and not broader publications by the WHO. Therefore, it will be incorrect to make the point in the results section. However, we have added updated lines 152 to the section to bring clarity. It now reads as “……. the WHO guideline documents (included in this review) highlighted confusion in the use of terms”.  Additionally, in the broader publication while the use of the term postnatal is more frequent, the two terms are still used interchangeably. |
| 4 | Just some additional points about the US for you to consider: Medicaid extension (public insurance) has been recently changed to include the postpartum period up to one year of giving birth and the Pregnancy Mortality Surveillance System (PMSS) defines a pregnancy-related death as a death while pregnant or within 1 year of the end of pregnancy from any cause related to or aggravated by the pregnancy. For maternal deaths in the US - the time frame is extended up to one year. You may want to consider adding documents that focus on capturing maternal deaths as they too might different in how the length of the postpartum period is defined. (https://www.hhs.gov/about/news/2023/12/15/hhs-to-improve-maternal-health-outcomes-with-new-cms-care-model-that-expands-access-to-services-other-proven-maternal-health-approaches.html) | Thank you for the suggestions. We highlighted these facts in lines 299-305, where we discuss about the late maternal deaths (maternal deaths after 42 days of delivery until completion of 1 year). This rise in ‘late’ maternal deaths prompt us to suggest an additional health contact after 42 days of delivery (4-6 months). We have also now included the recent development in the USA (lines 308-310) as you have suggested.  However, during the review, we observed that countries adhere to the WHO’s maternal deaths classification “the death of a woman while pregnant or within 42 days of termination of pregnancy, irrespective of the duration and the site of the pregnancy, from any cause related to or aggravated by the pregnancy or its management, but not from accidental or incidental causes” including the USA. The latest statistics published by the CDC (03/2023) have adhered to the WHO classification of maternal deaths despite the discussion on extension of financial cover up to one year (<https://www.cdc.gov/nchs/data/hestat/maternal-mortality/2021/maternal-mortality-rates-2021.pdf> ).  Australia- (11/2023) <https://www.aihw.gov.au/reports/mothers-babies/maternal-deaths-australia>  UK- (10/2023) <https://www.npeu.ox.ac.uk/mbrrace-uk/data-brief/maternal-mortality-2019-2021>  Canada (11/2019) <https://www150.statcan.gc.ca/n1/daily-quotidien/191126/dq191126c-eng.htm> |
| 5 | Around line 235 - I would suggest extending this paragraph to talk about the timeline used by countries to measure maternal mortality since this time frame also varies from 42 days by WHO to up to one year in the US. | The global morbidity and mortality reporting system – the International Classification of Diseases (ICD) a WHO project, defines the maternal mortality as deaths within 42 days of delivery (<https://icd.who.int/browse11/l-m/en#/http%3a%2f%2fid.who.int%2ficd%2fentity%2f1151689960> ). Therefore, globally for reporting purposes, this 42-day period is used. Despite the discussion on late maternal deaths (also defined in ICD <https://icd.who.int/browse11/l-m/en#/http%3a%2f%2fid.who.int%2ficd%2fentity%2f805843993> ) is gaining significant attention lately (We have highlighted it in our discussion (lines 299-305)), the length of 42 days is accepted and followed by countries, globally (as noted through the documents/links provided in the previous point). |
| 6 | Line 278 - how did you decide on 4-6 months here? | We acknowledge your concern and have further described the evidence supporting our argument.  Here, we put forward our recommendation first (additional health contact at 4-6 months) and then followed up it with the supporting evidence, in addition to the supportive facts presented before in the “discussion”.  Contact(s) between six and 12 months in addition to proposed four to six months contact will also be useful. However, some countries may find it difficult to resource many additional contacts (lines 287-288). Therefore, we propose addition of a contact with health services at four to six months based on the need,   1. To provide refresher/reminder on contraception, weight management, and birth spacing (lines 288-290) because birth spacing less than 6 months is associated with an increase in maternal and newborn complications (lines 251-255). 2. For screening and providing support for postpartum depression and excessive weight retention post-delivery, both of which are on the rise globally including low- and middle-income countries (lines 290-294). This is in line with the American Academy of Pediatrics recommendation for a follow up of mother at both 4 months and 6 months to screen for postpartum depression ( already indicate in lines 296 & 297). |
| 7 | Line 284 - In the US ACOG suggests a contact with the client within the first three weeks postpartum. (https://www.acog.org/clinical/clinical-guidance/committee-opinion/articles/2018/05/optimizing-postpartum-care) | Yes, we agree. We have already included it in table 1, last row (pg. 12) .  However, in this discussion section, we were highlighting the importance of extending the routine maternal care after 42 days to 4- 6 months. Therefore, we included the American Academy of Pediatrics recommendation of follow up of mother at both 4 months and 6 months to screen for postpartum depression. Thus, we did not highlight the care before 42 days in Discussion. |

We hope our responses and changes are to the satisfaction of the reviewers. We look forward to a positive response.

Sincerely,

Kiran Ahuja
