# Supplementary material for: Postpartum versus postnatal period: Do the name and duration matter?
Source: PLoS One. 2024 Apr 26;19(4):e0300118. doi: 10.1371/journal.pone.0300118 (PMC11051636; doi:10.1371/journal.pone.0300118)
Supplement: Attachment — Submitted filename: response to reviewers.docx [file pone.0300118.s004.docx]

19th February 2024

Douglas Aninng Opoku

Academic Editor

PLOS ONE

Dear Mr Opoku

We were pleased to have an opportunity to revise our manuscript titled “Postpartum versus postnatal period: Do the name and duration matter?” (PONE-D-23-34077R1). A point-by-point response to each of the comments raised is included in the Table below. Additionally, we have submitted a file with track changes and a clean copy of the revised documents.

|  | **Comment** | **Response** |
| --- | --- | --- |
| 1 | Indicate the type of review | We have included “Scoping review” to the Abstract (line 35) and the Materials and Methods (line 92) sections. |
| 2 | Indicate the time frame of the search (date, from when to when) | There was no start date – we included all documents available online published before 31 May 2023. This information has now been added to Abstract (line 35) and Materials and Methods section (line 91). |

Thank you for your consideration.

Sincerely,

[Kiran DK Ahuja](https://discover.utas.edu.au/Kiran.Ahuja), PhD

Senior Research Fellow, School of Health Sciences
